# Statistical significance: *p* value, 0.05 threshold, and applications to radiomics—reasons for a conservative approach

**DOI:** 10.1186/s41747-020-0145-y

**Published:** 2020-03-11

**Authors:** Giovanni Di Leo, Francesco Sardanelli

**Affiliations:** 1 Radiology Unit, IRCCS Policlinico San Donato, Via Morandi 30, 20097 San Donato Milanese, Italy; 2 Dipartimento di Scienze Biomediche per la Salute, Università degli Studi di Milano, Via Morandi 30, 20097 San Donato Milanese, Italy

**Keywords:** Confidence intervals, Decision making, Models (statistical), Radiomics, Reproducibility of results

## Abstract

Here, we summarise the unresolved debate about *p* value and its dichotomisation. We present the statement of the American Statistical Association against the misuse of statistical significance as well as the proposals to abandon the use of *p* value and to reduce the significance threshold from 0.05 to 0.005. We highlight reasons for a conservative approach, as clinical research needs dichotomic answers to guide decision-making, in particular in the case of diagnostic imaging and interventional radiology. With a reduced *p* value threshold, the cost of research could increase while spontaneous research could be reduced. Secondary evidence from systematic reviews/meta-analyses, data sharing, and cost-effective analyses are better ways to mitigate the false discovery rate and lack of reproducibility associated with the use of the 0.05 threshold. Importantly, when reporting *p* values, authors should always provide the actual value, not only statements of “*p* < 0.05” or “*p* ≥ 0.05”, because *p* values give a measure of the degree of data compatibility with the null hypothesis. Notably, radiomics and big data, fuelled by the application of artificial intelligence, involve hundreds/thousands of tested features similarly to other “omics” such as genomics, where a reduction in the significance threshold, based on well-known corrections for multiple testing, has been already adopted.

## Key points

- The *p* value reflects the degree of data compatibility with the null hypothesis.
- Some recommend abandoning *p* value, others lowering the significance threshold to 0.005.
- A 0.005 threshold could increase sample sizes and costs as well as depress spontaneous research.
- Authors should provide actual *p* values, not just “*p* < 0.05” or “*p* ≥ 0.05”.
- Threshold adjustments are needed for artificial intelligence-fuelled radiomics and big data.

## Background

A hot debate is long going on in major journals about *p* value and statistical significance. On the one side, those who “rise up against statistical significance”, as did Amrhein et al. in *Nature* [1]; on the other side, those who recommend “do not abandon significance”, as did Ioannidis in *JAMA* [2]. These are two opposite viewpoints around a long-standing topic: the misuse of *p* value and the claim of statistical significance or non-significance.

The *p* value is probably the most ubiquitous and, at the same time, misunderstood index in all of biomedical research. Hundreds of articles were published on this topic since at least the 1940s [3], with proposals of alternatives to the classic *p* value with its historical 0.05 threshold. One of these proposals, which has gained some attention, came in 2018 from Benjamin et al. [4], who suggested to lower the threshold for statistical significance from 0.05 to 0.005. However, despite this debate, clinical researchers still make large use of the classic 0.05 threshold.

In this article, we discuss the *value of p value* and explain why it should not be abandoned nor should the conventional threshold of 0.05 be modified. In addition, we show how artificial intelligence applications to radiomics and big data, involving hundreds/thousands of tested features, prompt reductions in the significance threshold, based on well-known corrections for multiple testing.

### Opposite opinions on the *p* value

In 2016, the American Statistical Association (ASA) released a statement warning against the misuse of statistical significance and *p* values [5]. A special issue in *The American Statistician* [5] presented 37 papers on “Statistical inference in the 21st century: a world beyond *p* < 0.05”.

The main ASA points are highlighted in the form of *do nots*, as follows:

- Do not base your conclusions solely on whether an association or effect was found to be *statistically significant* (*i.e*., the *p* value passed some arbitrary threshold such as *p* < 0.05);
- Do not believe that an association or effect exists just because it was statistically significant;
- Do not believe that an association or effect is absent just because it was not statistically significant;
- Do not believe that your *p* value gives the probability that chance alone produced the observed association or effect or the probability that your test hypothesis is true;
- Do not conclude anything about scientific or practical importance based on statistical significance (or lack thereof).

According to Wasserstein et al. [6], the statement *statistically significant* has today become meaningless. In 1885, Edgeworth’s original intention for statistical significance was simply to have a tool to indicate when a result warrants further scrutiny; statistical significance was never meant to imply *scientific importance*, but that idea has been irretrievably lost [7]. Yet a century later, the confusion persists. Such doubts can lead to radical choices, such as the one taken by the Editors of *Basic and Applied Social Psychology*, who decided to ban *p* values in 2015 [8].

Wasserstein et al. [6] also say that “no *p* value can reveal the plausibility, presence, truth, or importance of an association or effect”. Therefore, a label of statistical significance does not mean or imply that an association or effect is highly probable, real, true, or important, nor “does a label of statistical nonsignificance lead to the association or effect being improbable, absent, false, or unimportant”. Furthermore, this false split into *worthy* and *unworthy* results leads to the selective reporting and publishing of results based on their statistical significance, the so-called *publication bias*. Similarly, Wasserstein et al. [6] also suggest to stop using confidence intervals (CIs) as another means of dichotomisation, based on whether a *null value* falls within the interval. However, despite these considerations, the ASA does not recommend to stop the *p* value calculation. The two sides of the debate may be conciliated. Just, when *p* values are used, they should be reported as continuous quantities, not claiming significance or non-significance. The ASA position may be summarised with the sentence “as statistical significance is used less, statistical thinking will be used more”.

Opposite to the ASA position is that of Ioannidis [2] and other authors [9, 10] who exhort not to abandon statistical significance. We agree with this viewpoint. We acknowledge the importance of embracing uncertainty, avoiding hyped claims, and recognising that the *p* value is often poorly understood, but statistical significance, in our opinion, has a crucial practical importance. Inferences are unavoidably dichotomous, especially in medicine and healthcare, both in preclinical (*experimental*) and clinical research. Any intervention, such as a new drug or imaging technique, will either be licensed or not. As such, *we do need a methodology that allows for dichotomisation*. Although the policymakers base their decisions also on other factors (*e.g.*, economics), scientific evidence with roots into statistical significance should help in doing so. We believe that there is a need for inductive thinking and statistical tools to support inferences. If the *bar* would be removed, any difference may be claimed to reflect an important effect. In front of results from a well-conducted study not using dichotomisation, policymakers would ask “So, what?”.

On the side of those favouring the use of *p* value and its dichotomisation, Benjamin et al. [4] and Ioannidis [11] rather proposed to lower the significance threshold from the conventional 0.05 to 0.005. This proposal comes essentially with the aim to reduce the false discovery rate, *i.e*., the rate of claims of associations or effects that are later not replicated. According to Ioannidis [11], “the reduction in the *p* value threshold may largely do more good than harm, despite also removing an occasional true and useful treatment effect from the coveted significance zone”. In our opinion, the reduction of the threshold to one tenth of the current one has more disadvantages than advantages, at least for clinical research.

### The threshold for significance and its origin

The above-mentioned five points from ASA are surely relevant. To better understand their meaning, we need to place them into the historical context. In the theoretical system proposed by Ronald A. Fisher (1890–1962), the *p* value had to be considered only as a rough guide of the strength of evidence against the null hypothesis. In other words, the meaning of *p* < 0.05 was merely that one should repeat the experiment. If subsequent studies also yielded significant *p* values, one could conclude that the observed effects were unlikely to be solely the result of chance.

For decades, 0.05 (5%, *i.e*., 1 of 20) has been conventionally accepted as the threshold to discriminate significant from non-significant results, inappropriately translated into existing from not existing differences or phenomena. This cutoff has peculiar reasons. Early in the 1900s, statistics textbooks reported many tables with long series of *p* values. Fisher shortened the tables previously published by Karl Pearson (1857–1936), not only for reasons of editorial space but probably also for copyright reasons (it seems that Fisher and Pearson were not on good terms). Some *p* values were selected and became more important than others, as Fisher wrote for researchers (the users) and not for expert in statistics (the theoreticians). Fisher himself provided a selection of probabilities which simplified the choice to help in decision-making [12] and attributed a special status to 0.05, asserting explicitly that “the value for which *p* = 0.05, or 1 in 20, is 1.96 or nearly 2. It is convenient to take this point as a limit in judging whether a deviation ought to be considered significant or not” [13].

Research methodology in medicine is such that a comparison between two or more datasets (groups) is typically performed in terms of a given endpoint. Examples include the comparison between the efficacy of a new drug/treatment *versus* the established drug/treatment or placebo in two different animal or human population samples, or the comparison between the diagnostic accuracy of different imaging techniques. For a reason or another, a study commonly comes up with different values for the measured endpoints in two (or more) groups, and researchers need to ascertain if the observed difference is actually due to random sampling or, instead, reflects a real difference among the groups [14].

These comparisons are typically carried out through one or more statistical tests. Briefly, statistical tests are based on the following question: *if there is not an existing difference between the two compared groups*, *what is the probability to obtain the observed difference or a larger one that is only due to random sampling*? Being the answer to this question a probability in nature, the famous *p* value, it is never 1 (100%) or 0 (0%), but always something in the middle. As such, it does not provide the probability for the null hypothesis (*i.e*., that there is not a real difference between the two groups) to be true, but rather reflects the *degree of compatibility* of the data with the null hypothesis. Thus, the *decision to reject* the null hypothesis must necessarily be based on a threshold defined a priori. In practice, the smaller the calculated *p* value, the more we *consider* the null hypothesis to be improbable; consequently, the smaller the *p* value, the more we *consider* the alternative hypothesis to be probable (*i.e.*, that the groups are indeed different) [14].

### The threshold: to lower or not to lower?

Ioannidis based his proposal to reduce the threshold to 0.005 on a previous article in *PLoS Medicine* [15] presenting a theoretical framework relating the post-study probability of a research hypothesis to be true to the pre-study probability. The author dramatically stated that “most published research claims are false”, thus receiving extensive attention. Goodman and Greenland [16] have already argued that “the mathematical argument in the *PLoS Medicine* paper underlying the proof of the title’s claim has a degree of circularity” and that “the claims that the model employed in this paper constitutes a proof that most published medical research claims are false […] are unfounded”.

According to Ioannidis [11], “moving the *p*-value threshold from .05 to .005 will shift about one-third of the statistically significant results of past biomedical literature to the category of just suggestive”. We think that such a solution makes biomedical research harder and that, adopting this solution, an improvement in research quality is not granted. Lowering this way the *p* value threshold for significance is, at best, a palliative solution. Especially in clinical research, future trials would need to be larger, less feasible, and more expensive. Achieving 80% power with a threshold of 0.005, instead of 0.05, would require a 70% larger sample size for between-subject study designs with two-sided tests (88% for one-sided tests) [17]. Researchers could abandon some good ideas with the net effect of depressing spontaneous, investigator-initiated research. Only the few treatments with large effect sizes would gain the evidence, thanks to the statistical power granted by medical industries. Conversely, treatments with a small yet clinically appreciable effect would be hardly proven as effective. This would be amplified in studies where the outcome of interest is quite infrequent such as interval cancers in breast cancer screening mammography, cardiovascular events, or cancer recurrence in longitudinal studies. Not to mention rare diseases or those studies where the tested treatment or diagnostic tool is invasive and poses ethical or organisational issues.

These arguments are not new. An article signed by 54 authors [18] has provided a similar view, with a deeper technical explanation on why the statements by Ioannidis [15] and Benjamin et al. [4] are unjustified. Another article, signed by 88 authors [17], has questioned the idea that the significance threshold should be based on the amount of relative evidence indicated by Bayesian factors^1^, as done by Benjamin et al. [4], whose assumptions were considered to be unjustified.

A major point deserving further comments is the lack of replicability/reproducibility of study results [4]. Causes of this include multiple testing, *p*-hacking, publication bias, and underpowered studies. As Gregg Easterbrook said, “Torture numbers, and they will confess to anything” (https://todayinsci.com/E/Easterbrook_Gregg/EasterbrookGregg-Quotations.htm). A famous example of non-replication came in 2006, when a group of researchers presented an algorithm using genomic microarray data that predicted cancer patients being responders to chemotherapy [19]. This paper drew immediate attention. Two statisticians later obtained the publicly available data and attempted to apply the algorithm [20]. What they found was a very poorly conducted data analyses, with errors ranging from trivial to devastating. It was not until 2011 that the original study was retracted from *Nature Medicine*.

Interestingly, the probability to replicate a study showing significant results has been estimated to be only 62–67% for a statistical power of 80%, or 69–76% for a statistical power of 90% [21]. To note, 2/3 (*i.e.,* 66.7%) “is the probability Laplace derived for repeating a successful event when the first event emerged against a background of perfect ignorance” [21].

To better understand the impact of lowering the significance threshold from 0.05 to 0.005, we show some examples of articles leading to 0.005 < *p* < 0.05 that were later considered as relevant evidence guiding clinical practice.

### Examples of studies leading to 0.005 < *p* < 0.05

From the table of evidence in support of the recommendations of the 2019 ACC/AHA Guideline on the primary prevention of Cardiovascular Disease–A Report of the American College of Cardiology/American Heart Association Task Force on Clinical practice Guidelines [22], we extracted the two following examples (overall, in this guideline, there are dozens of original articles relying on a 0.005 < *p* < 0.05). The Multi-Ethnic Study of Atherosclerosis [23] demonstrated, among other things, that early menopause (below 46 years) is a moderate independent predictor of coronary heart disease and stroke in a diverse population of women in the USA. In particular, the incidence of hard coronary events was 7.33/1,000/year in women with early menopause and 3.22/1,000/year in women without, for a fully adjusted hazard ratio (HR) of 1.85 (95% CI 1.01–3.37), *p* = 0.045. The fully adjusted HR for stroke was 2.03 (95% CI 1.00–4.10), *p* = 0.049. Notably, the study prospectively enrolled 2,509 women but would have had to enrol about 4,265 women (2,509 increased by 70%; see ref [17]) had the significance threshold lowered to 0.005, with inherent higher costs and time needed to complete the study. The Women’s Health Initiative Observational Study [24] demonstrated that among apparently healthy subjects aged 50–79, sitting time ≥ 10 h/day (*versus* ≤ 5 h/day) was moderately associated with risk of coronary heart disease, for an adjusted HR of 1.13 (95% CI 1.01–1.26), *p* = 0.04. For stroke, the HR was 1.18 (95% CI 1.04–1.34), *p* = 0.008.

From the European Society of Breast Imaging (EUSOBI) recommendations for women’s information on breast magnetic resonance imaging, we extracted the following example. The High Breast Cancer Risk Italian study [25] evaluated 501 women at high risk of breast cancer and demonstrated that the incidence in women with previous history of breast cancer (29/674, 4.3%, 95% CI 2.9–6.1%) was significantly higher than that in women without (23/918, 2.5%, 95% CI 1.6–3.7%), *p* = 0.045. As this population is hard to collect, the study involved 18 centres for more than 7 years. This would have been not feasible at all had the significance threshold set at 0.005.

The updated NICE guidelines on cardiac computed tomography as the first-line test for coronary artery disease were based, among other things, on the article by Williams et al. [26] to support recommendations. This randomised trial on 4,146 patients showed that when preventive therapies were implemented ≥ 50 days after computed tomography, the rate of fatal and non-fatal myocardial infarction was halved in the patients allocated to computed tomography (HR = 0.50; *p* = 0.020) [26]. Being this the result of a subgroup analysis, it could not be found had the significance threshold set at 0.005.

These few examples clearly show the difficulties in reaching statistical significance in clinical research. But similar problems are faced also in preclinical research on animal models, with specific ethical concerns [27]. In particular, the use of animal models should be discouraged and kept to the minimum, a criterion that is in contradiction with the need for a larger sample size following significance threshold reduction.

### Beyond the *p* value: secondary evidence and data sharing

Regardless of the misuse of *p* value and lack of reproducibility, too much importance is given to the *p* value threshold rather than to biases as well as selective reporting and non-transparency in published studies. There are many stages from the original idea to data analysis of a study, with the *p* value being the very last. Decisions that are made prior to discuss the *p* value have a greater impact on results, including design, lack of adjustment for confounding factors, and simple measurement errors. Biases may force significant findings to come out, with spurious effect sizes that are later rebutted. To a certain degree, biases may lead to a *p* value lower than any threshold. As acknowledged by Ioannidis [11], malicious researchers would easily avoid the obstacle by defining, perhaps a posteriori, weak surrogate endpoints. Yet, simply reducing the significance threshold probably would not attenuate these problems.

More importantly, healthcare policymakers typically base their decisions on secondary evidence, such as systematic reviews and meta-analyses or cost-effectiveness analyses, which summarise the available evidence taking into consideration the methodological quality of the analysed studies. Systematic reviews “dismiss mostly the noise” referred to by Ioannidis [11]. Policymakers do not usually take decisions based on a single study of few patients reaching *p* just below the 0.05 threshold. We should never forget that “science is built up of facts, as a house is with stones. But a collection of facts is no more a science than a heap of stones is a house”, as Jules Henri Poincaré said (https://www.brainyquote.com/quotes/henri_poincare_164238).

As said by Steven Goodman, “there is no number generated by standard methods that tells us the probability that a given conclusion is right or wrong”. Efforts should be instead paid to the researchers’ training and to policies that may further improve research quality, such as a priori registration of a trial protocol, the need for a professional statistician for data analysis, and data sharing [6]. Especially, data sharing has the potential for verification by independent authors of the results presented in a given publication [28]. When data are shared, they may be used by other researchers to perform alternative or supplementary analyses. An independent analysis may show results in support of the initial findings or could instead reveal errors or inconsistencies in the original research. Finally, data sharing could potentially lead to an optimisation of time and costs of clinical research by preventing the duplication of trials.

### The peculiar case of radiomics

Concerns on the so-called *multiple testing burden* hold in any research dealing with numerous variables and significance thresholds must be adapted according to established methods. For example, genome-wide association studies (GWAS) are such that millions of statistical tests are typically performed. Keeping the significance threshold at the conventional value of 0.05 would lead to a large number of false-positive results. For example, if 1,000,000 tests are carried out, then 5% of them (that is, 50,000 tests) are expected to lead to *p* < 0.05 by chance when the null hypothesis is actually true for all these tests. The multiple testing burden has led to the adoption of stringent significance thresholds in GWAS, such as 5 × 10^−8^, reflecting a Bonferroni correction for the one million independent tests performed in a GWAS [29, 30]. Application of this threshold has increased the robustness and reproducibility of claimed associations [31]. However, it is important to highlight that this lowered threshold actually derives from the conventional 0.05 threshold after the Bonferroni correction [29, 30], not from an arbitrary reduction. Further details can be found in the review by Sham and Purcell [32].

The integration into clinical research of enormous datasets as typically happens for radiomics may determine a situation not different from that of genomics. Radiomics is defined as the extraction of a large number of quantitative features from medical images, with the distinct advantage of assessing tissue heterogeneity. Radiomic features offer quantitative measurements of tissues through three-dimensional images including texture, intensity, heterogeneity, and morphology information allowing a comprehensive phenotype analysis.

Radiomics may involve a high number of statistical tests, with a potential for a high false discovery rate. In a typical radiomic study, hundreds to thousands of features can be extracted to be correlated to different outcomes. The number of features is often greater than the number of analysed patients (the so-called *large p*, *small n problem*), leading to false-positive results and overfitting [33, 34]. For these studies, the significance threshold should be corrected for multiple hypotheses testing using the Bonferroni method or a false discovery rate controlling procedure, such as the Benjamini-Hochberg method [34]. Interestingly, searching for the optimal threshold by keeping the false discovery rate at a desired level (*e.g*., ≤ 5%) or using the Bonferroni method both yield overly conservative values for threshold [35]. Moreover, as the optimal threshold can vary in different datasets, the results may not be generalizable. Especially for survival analysis, selection of an optimal threshold is not recommended [34, 36]. Anyway, regardless of the significance threshold, false discoveries in radiomics can be identified by external validation on at least one large independent dataset, an essential condition for declaring any diagnostic or prognostic value of machine/deep learning applications to clinical imaging [37].

### Alternatives to the *p* value

Several alternatives to the *p* value have been proposed. A comprehensive discussion is beyond this article’s aim; further details may be found in Wasserstain et al. [6]. Briefly, proposals coming from the *frequentist school* go all around a hybrid combination of *p* value, meaningful/clinically important effect size, 95% CIs, and other metrics. On the other side, the *Bayesian school* offers the use of the so-called Bayesian factors, with inherent strengths and weaknesses. The Bayesian school is based on the Bayes’ theorem (also called theorem of conditioned probability), for which the probability that the result of an examination is associated with the presence or the absence of the disease depends on the pre-examination probability and the power of the examination. As a matter of fact, none of these methods have really seen a widespread use in clinical research.

To this regard, a simulation study [38] has shown some cornerstones, summarised as follows:

1. The *p* value-only approach to inferential statistics for studies that are properly powered is associated to the highest false discovery rate (63% or 47% when 0.05 or 0.005 are used as the significance threshold, respectively).
2. The effect size-only method falters in terms of false discovery rate (43%) for low-power studies, or when the effect size is comparatively small.
3. A method that combines *p* values with the effect size can generate heuristic, but non-definitive evidence.

In summary, all of the many methods compared in the study by Goodman et al. [38] have strengths and weaknesses and none of them generates an automatic final answer for a definitive inference. These methods are not so much different to one another and often provide similar interpretations of uncertainty [39]. However, there are cases where these statistical methods do not clearly align. Closer inspection of these cases likely reveals problems with sample size, study design, or implementation of protocols.

An interesting advancement is the so-called *second-generation p value*, based on an *expanded null hypothesis* [40]. The idea is to use a composite null hypothesis that takes into consideration the limits of experimental precision in outcome measurement as well as the clinical relevance. The null interval should contain, in addition to the precise point null hypothesis, all other points that are practically/clinically equivalent. An example of interval null hypothesis for an odds ratio (OR) may be H_0_ 0.95 ≤ OR ≤ 1.05 instead of H_0_ OR = 1, as typically done in clinical research. In other words, rejecting the null hypothesis that two effects are identical is not helpful, because they could still be nearly identical for all practical purposes. Indeed, this method resembles that behind the well-known non-inferiority studies.

## Educational issues and conclusions

The use of *p* value and its dichotomisation remains a matter for debate. As said by Krueger and Heck [21], “hearing that *p* values are terrible and that, by the way, they are not low enough recalls the vacationer’s complaint that The food was horrible – and the portions were so small!”. The two complaints nullify each other. In our opinion, the general conclusion that the *p* value has no evidentiary value at all seems overstated. It is difficult to argue that there is no difference between *p* = 0.8 and *p* = 0.08. A *p* value lower than 0.05 does not ensure that the result is replicable, but simulations show that one may be guardedly optimistic about replication. Yet, a replication study is itself no more decisive than the original study, but each additional study provides a small incremental contribution to the cumulative evidence. We do not recommend to abandon the *p* value, nor to reduce arbitrarily the threshold for significance. A reduced threshold would add confusion to the existing confusion and would have negative impacts on research, especially depressing spontaneous not industry-driven projects.

One reason why *p* value persists is that it is part of the vocabulary of research. The scientific community feels they understand the rules and is generally not familiar enough with alternative methodologies. This was discovered by the Editor of *Epidemiology*, who tried to ban the use of *p* value but was forced to abandon the idea after several years [41].

All this matter highlights important educational issues [14]. First, to reinforce the *recommendation for authors to always report real p values as continuous quantities*, *not as* “≥ 0.05” or “< 0.05”. In other words, any declaration of significance or non-significance must be given with an exact *p* value. Second, *attention has to be paid to multiple testing and its consequences in terms of threshold reduction*, especially for the emergent application of artificial intelligence to radiomics, following the example of genomics. Last but not least, researchers and readers must always remember the following: (1) *a result can be highly statistically significant but completely clinically irrelevant*, as is when a new drug prolongs patient survival by few weeks or a new imaging technique increases the accuracy by a very small amount (the effect size is too small); (2) *a not significant result can be highly clinically relevant*, *posing doubt about the statistical power of the study*, as is when a new drug prolongs patient survival by several months or a new imaging technique increases the accuracy by 20%. The lack of preliminary sample size calculation in many radiological studies shows that a lot of educational work is needed.

Research quality cannot be improved by merely abandoning the concept of statistical significance or simply increasing the prize for it, reducing the threshold. Disadvantages of the current 0.05 thresholds are mitigated by secondary evidence that commonly guides medical decision-making. After all, if biomedical research has reached the level where it is now, it is unlikely that most of it was false.

## Data Availability

Not applicable

## Abbreviations

ASA: American Statistical Association
CI: Confidence interval
GWASs: Genome-wide association studies
HR: Hazard ratio

## Footnotes

1 See the “Alternatives to the *p* value” section for a short explanation of the Bayesian school.

## References

[1] Amrhein V, Greenland S, McShane B (2019). Scientists rise up against statistical significance. Nature.

[2] Ioannidis JPA (2019). The importance of predefined rules and prespecified statistical analyses: do not abandon significance. JAMA.

[3] Berkson J (1942). Tests of significance considered as evidence. J Am Stat Assoc.

[4] Benjamin DJ, Berger JO, Johnson VE (2018). Redefine statistical significance. Nat Hum Behav.

[5] Wasserstein RL, Lazar NA (2016). The ASA’s statement on p-values: context, process, and purpose. Am Stat.

[6] Wasserstein RL, Schirm AL, Lazar NA (2019) Moving to a world beyond “*p*<0.05”. Am Stat 73:1–19. 10.1080/00031305.2019.1583913

[7] Boring EG (1919). Mathematical vs. scientific significance. Psychol Bull.

[8] Trafimow D, Marks M (2015). Editorial. Basic Appl Soc Psyc.

[9] Leek JT, Peng RD (2015). Statistics: p-values are just the tip of the iceberg. Nature.

[10] Nuzzo R (2015) Scientists perturbed by loss of stat tool to sift research fudge from fact. Sci Am. https://www.scientificamerican.com/article/scientists-perturbed-by-loss-of-stat-tools-to-sift-research-fudge-from-fact/. Accessed May 2, 2019

[11] Ioannidis JPA (2018) The proposal to lower P value thresholds to .005. JAMA 319:1429–1430. 10.1001/jama.2018.1536 10.1001/jama.2018.1536 29566133

[12] Soliani L (2007) Statistica applicata alla ricerca e alle professioni scientifiche. Manuale di statistica univariata e bivariata. Uninova-Gruppo Pegaso, Parma, pp 8–11 http://www.dsa.unipr.it/soliani/soliani.html. Accessed May 2, 2019

[13] Fisher RA (1956). Statistical methods for research workers.

[14] Sardanelli F, Di Leo G (2009) Biostatistics for radiologists: Planning, performing, and writing a radiologic study. Springer-Verlag, Milan, pp 68–71

[15] Ioannidis JPA (2005). Why most published research findings are false. PLoS Med.

[16] Goodman S, Greenland S (2007). Why most published research findings are false: problems in the analysis. PLoS Med.

[17] Lakens D, Adolfi FG, Albers CJ (2018). Justify your alpha. Nat Hum Behav.

[18] Trafimow D, Amrhein V, Areshenkoff CN (2018). Manipulating the alpha level cannot cure significance testing. Front Psychol.

[19] Potti A, Dressman HK, Bild A (2011). Retraction: genomic signatures to guide the use of chemotherapeutics. Nat Med.

[20] Baggerly KA, Coombes KR (2009). Deriving chemosensitivity from cell lines: forensic bioinformatics and reproducible research in high-throughput biology. Ann Appl Stat.

[21] Krueger JI, Heck PR (2017). The heuristic value of p in inductive statistical inference. Front Psychol.

[22] Arnett DK, Blumenthal RS, Albert MA (2019). 2019 ACC/AHA Guideline on the primary prevention of cardiovascular disease: executive summary: a report of the American College of Cardiology/American Heart Association Task Force on Clinical Practice Guidelines. J Am Coll Cardiol.

[23] Wellons M, Ouyang P, Schreiner PJ, Herrington DM, Vaidya D (2012). Early menopause predicts future coronary heart disease and stroke: the Multi-Ethnic Study of Atherosclerosis. Menopause.

[24] Chomistek AK, Manson JE, Stefanick ML (2013). Relationship of sedentary behavior and physical activity to incident cardiovascular disease: results from the Women’s Health Initiative. J Am Coll Cardiol.

[25] Sardanelli F, Podo F, Santoro F (2011). Multicenter surveillance of women at high genetic breast cancer risk using mammography, ultrasonography, and contrast-enhanced magnetic resonance imaging (the high breast cancer risk Italian 1 study): final results. Invest Radiol.

[26] Williams MC, Hunter A, Shah AS (2016). Use of coronary computed tomographic angiography to guide management of patients with coronary disease. J Am Coll Cardiol.

[27] Ferdowsian HR, Gluck JP (2015). The ethical challenges of animal research. Camb Q Healthc Ethics.

[28] Sardanelli F, Alì M, Hunink MG, Houssami N, Sconfienza LM, Di Leo G (2018). To share or not to share? Expected pros and cons of data sharing in radiological research. Eur Radiol.

[29] Pe’er I, Yelensky R, Altshuler D, Daly MJ (2008). Estimation of the multiple testing burden for genomewide association studies of nearly all common variants. Genet Epidemiol.

[30] Jannot AS, Ehret G, Perneger T (2015). P < 5 × 10(-8) has emerged as a standard of statistical significance for genome-wide association studies. J Clin Epidemiol.

[31] Welter D, MacArthur J, Morales J (2014). The NHGRI GWAS Catalog, a curated resource of SNP-trait associations. Nucleic Acids Res.

[32] Sham PC, Purcell SM (2014). Statistical power and significance testing in large-scale genetic studies. Nat Rev Genet.

[33] Alic L, Niessen WJ, Veenland JF (2014). Quantification of heterogeneity as a biomarker in tumor imaging: a systematic review. PLoS One.

[34] Chalkidou A, O’Doherty MJ, Marsden PK (2015). False discovery rates in PET and CT studies with texture features: a systematic review. PLoS One.

[35] Hilsenbeck S, Clark G, McGuire W (1992). Why do so many prognostic factors fail to pan out?. Breast Cancer Res Treat.

[36] Altman DG, Lausen B, Sauerbrei W, Schumacher M (1994). Dangers of using “optimal” cutpoints in the evaluation of prognostic factors. J Natl Cancer Inst.

[37] Pesapane F, Codari M, Sardanelli F (2018). Artificial intelligence in medical imaging: threat or opportunity? Radiologists again at the forefront of innovation in medicine. Eur Radiol Exp.

[38] Goodman WM, Spruill SE, Komaroff E (2019) A proposed hybrid effect size plus p-value criterion: empirical evidence supporting its use. Am Stat 73(suppl 1):168–185. 10.1080/00031305.2018.1564697

[39] Wetzels R, Matzke D, Lee MD, Rouder JN, Iverson GJ, Wagenmakers J (2011). Statistical evidence in experimental psychology: an empirical comparison using 855 t tests. Perspect Psychol Sci.

[40] Blume JD, Greevy RA, Welty VF, Smith JR, Dupont WD (2019) An introduction to second-generation p-values. Am Stat 73:sup1:157–167. 10.1080/00031305.2018.1537893

[41] Lang JM, Rothman KJ, Cann CI (1998) That confounded p-value. Epidemiology 9:7–8. 10.1097/00001648-199801000-00004 10.1097/00001648-199801000-00004 9430261

